# Gene Set Enrichment Analyses: lessons learned from the heart failure phenotype

**DOI:** 10.1186/s13040-017-0137-5

**Published:** 2017-05-26

**Authors:** Vinicius Tragante, Johannes M. I. H. Gho, Janine F. Felix, Ramachandran S. Vasan, Nicholas L. Smith, Benjamin F. Voight, CHARGE Heart Failure Working Group, Colin Palmer, Pim van der Harst, Jason H. Moore, Folkert W. Asselbergs

**Affiliations:** 1 Department of Cardiology, Division Heart & Lungs, University Medical Center Utrecht, Heidelberglaan 100, 3584 CX Utrecht, The Netherlands; 2 Department of Epidemiology, Erasmus MC, University Medical Center Rotterdam, Rotterdam, The Netherlands; 3 Departments of Medicine and Preventive Medicine, Boston University School of Medicine, Boston, MA USA; 4 Department of Epidemiology, University of Washington, Seattle, WA USA; 5 Department of Systems Pharmacology and Translational Therapeutics, Perelman School of Medicine, University of Pennsylvania, Philadelphia, PA USA; 6 Department of Genetics, Perelman School of Medicine, University of Pennsylvania, Philadelphia, PA USA; 7 Institute for Translational Medicine and Therapeutics, Perelman School of Medicine, University of Pennsylvania, Philadelphia, PA USA; 8 Population Pharmacogenetics Group, University of Dundee, Dundee, UK; 9 Department of Cardiology, University Medical Center Groningen, University of Groningen, Groningen, The Netherlands; 10 Department of Biostatistics and Epidemiology, Institute for Biomedical Informatics, Perelman School of Medicine, University of Pennsylvania, Philadelphia, PA USA; 11 Durrer Center for Cardiovascular Research, ICIN-Netherlands Heart Institute, Utrecht, The Netherlands; 12 Institute of Cardiovascular Science, Faculty of Population Health Sciences, University College London, London, UK; 13 Farr Institute of Health Informatics Research and Institute of Health Informatics, University College London, London, UK

**Keywords:** Gene set enrichment analyses, Heart failure, Coronary artery disease

## Abstract

**Background:**

Genetic studies for complex diseases have predominantly discovered main effects at individual loci, but have not focused on genomic and environmental contexts important for a phenotype. Gene Set Enrichment Analysis (GSEA) aims to address this by identifying sets of genes or biological pathways contributing to a phenotype, through gene-gene interactions or other mechanisms, which are not the focus of conventional association methods.

**Results:**

Approaches that utilize GSEA can now take input from array chips, either gene-centric or genome-wide, but are highly sensitive to study design, SNP selection and pruning strategies, SNP-to-gene mapping, and pathway definitions. Here, we present lessons learned from our experience with GSEA of heart failure, a particularly challenging phenotype due to its underlying heterogeneous etiology.

**Conclusions:**

This case study shows that proper data handling is essential to avoid false-positive results. Well-defined pipelines for quality control are needed to avoid reporting spurious results using GSEA.

**Electronic supplementary material:**

The online version of this article (doi:10.1186/s13040-017-0137-5) contains supplementary material, which is available to authorized users.

## Introduction

Gene Set Enrichment Analysis (GSEA) is a statistical method to assess whether differences in expression of gene sets between two phenotypes are statistically significant [1, 2]. It was initially designed for analysis of mRNA expression values, obtained from the then recently developed microarray technology [3], based on the observation that existing methods at the time were not capable of separating the small difference in expression profiles between two classes, and a grouping strategy was necessary. The original study [3] also developed collections of gene sets, based on biological knowledge available at the time.

Since then, multiple additional methods and reference gene sets have been developed, in an attempt to tackle caveats that emerged with the increase in use of GSEA and the new types of data, such as genome-wide association studies (GWAS) and exome sequencing. Among the current existing methods, Pathway Studio [4], MAGENTA [5], PANTHER [6], EVA [7] and Ingenuity [8] are commonly used, whereas the most common gene set definitions are Gene Ontology [9], KEGG [10, 11], REACTOME [12, 13], BIOCARTA [14] and MSIGdb [15].

Heart failure (HF) is a major medical problem of the Western world, carrying a high morbidity, mortality and economic burden [16, 17]. The susceptibility to develop HF is thought to be partially genetically based [18], but despite a tremendous increase in knowledge regarding etiology and risk factors for HF, still relatively little is known about genetic factors related to HF incidence. Until now, genetic causes of HF have mainly been identified in rare cases of non-ischemic HF with monogenic inheritance [19]. Genetic studies for complex diseases, such as HF, focus on main effects of single loci using strict statistical thresholds for significance, and typically do not consider more complicated biological, genomic, or environmental hypotheses and models in their primary scans.

While genome-wide studies have succeeded in identifying a multitude of genetic variants affecting disease risk, for incident heart failure (HF) thus far only two SNPs have been identified in different ethnicities [20], most likely due to the relatively small sample sizes of the efforts so far, and the fact that HF is a very heterogeneous phenotype. Functional SNPs with small main effects may not replicate across studies due to context-dependent effects, such as the selection criteria of each cohort. Novel alternative analysis approaches to GWAS data that focus on the combined effects of many loci, each making a small contribution to overall disease susceptibility, such as GSEA, may provide a solution for the aforementioned limitations. Based on evidence from GSEA, SNPs may be selected for further studies even if the association of that SNP with heart failure is sub-genome-wide significant. Discovery of loci that contribute susceptibility to complex diseases like HF through gene-by-gene or gene-by-environment interactions may segregate main effects at the individual loci that are weak or even entirely absent, motivating approaches like GSEA or pathway-based methods that detect association at the biological systems level.

We hypothesize that multiple loci interact to contribute to development of HF. GSEA can be used to summarize genome-wide and exome array data integrating biochemical systems and gene function. With possible gene-gene interactions present in gene sets, potentially novel pathophysiological pathways can emerge, underlying the development of HF, which are missed by conventional methods.

Given the statistical approach of each method, the gene sets and the input each one takes, outputs obtained for a given phenotype may differ widely between GSEA methods, causing uncertainty on how to interpret the data and move research forward. Our goal in this paper is not to review each method separately (refer to Elbers et al. [21] for a broad comparison), but instead to offer general guidelines that can be applied to every method of the GSEA class. To illustrate these methods, we use genome-wide data from the CHARGE consortium [20], PREVEND [22, 23] and Go-DARTS [24] reporting incident HF, and CARDIoGRAM [25] and C4D [26] for coronary artery disease (CAD).

## Cohort descriptions

### Discovery

We use in this paper as input the results of the GWAS meta-analysis on incident heart failure performed by the CHARGE (Cohorts for Heart and Aging Research in Genomic Epidemiology) consortium [20]. The analysis of the CHARGE - Heart Failure Working Group, part of the CHARGE Consortium, included 4 prospective cohort studies: the Atherosclerosis Risk in Communities Study (ARIC), the Cardiovascular Health Study (CHS), the Framingham Heart Study (FHS) and the Rotterdam Study (RS). These studies included participants of European and African ancestry who were free of HF at study baseline. Incident HF cases were identified during follow-up by self-report, administrative data or periodic clinical in-study examinations. A total of 20,926 participants of European ancestry and 2895 participants of African ancestry with available genome wide data were eligible. The average age at baseline ranged from 53.3 to 72.6 years and 57% were women. In total 2526 (12.1%) incident HF events were identified among those of European ancestry over an average of 11.5 years follow-up and 466 (16.1%) during 13.7 years of follow up among those of African ancestry. The average age at the time of HF onset was 73.6 years and 52% of events occurred among women. In those with European and African ancestry, 73% and 78%, respectively, had no history of myocardial infarction prior to the diagnosis of heart failure.

## Replication

Firstly, we conducted replication using incident heart failure cases from the Genetics of Diabetes Audit and Research Study in Tayside Scotland (Go-DARTS) [24], a cohort with European individuals with type 2 diabetes, which was genotyped using Illumina ExomeChip. This chip gives us the advantage of having one-to-one SNP-to-gene mapping, because protein-coding SNPs were included in this chip, and their mapping is known. Secondly, we conducted replication using heart failure cases and GWAS data of the Prevention of REnal and Vascular ENdstage Disease (PREVEND) Study (*n* = 3,418, non-diabetics), a Dutch ongoing prospective study investigating the natural course of increased levels of urinary albumin excretion and its relation to renal and cardiovascular disease [22, 23].

All studies in CHARGE, Go-DARTS and PREVEND received institutional review board approval, and all participants provided written informed consent for the use of their DNA for research.

## Initial pathway results

We analyzed the CHARGE [20] meta-analysis results for HF with EVA [7] using MSIGdb [15]. We defined *P* < 0.05 as the threshold for significance, calculated via enrichment tests counting the number of SNPs below this threshold as compared to the total of SNPs mapped to each gene, in the first step. We mapped SNPs to genes using BEDtools [27], dbSNP142 and the RefSeq gene reference, both datasets downloaded from the UCSC Genome Browser [28], using a 500 kb window for mapping. We then used the obtained gene *p*-values to calculate pathway *p*-values, by counting the number of gene *p*-values below the significance threshold of 0.05 as a proportion of the number of genes in each pathway.

We followed exactly the same procedure and phenotype (HF) for the other two datasets (GoDARTS and PREVEND), with the exception of the SNP-to-gene mapping step for GoDARTS since all Exome chip SNPs are known to code specific genes. Additional file 1: Table S1 presents *p*-values for all significant pathways in the analysis of the CHARGE data and their *p*-values from the analyses of the other two cohorts.

Four pathways were significant for all three studies: KEGG_TYPE_I_DIABETES_MELLITUS, KEGG_ALLOGRAFT_REJECTION, KEGG_GRAFT_VERSUS_HOST_DISEASE and KEGG_ASTHMA. These pathways share a considerable amount of SNPs (15 out of 30, 43, 36 and 41, respectively), the vast majority of which is related to the MHC complex (Additional file 2: Figure S1). Permutation tests run in R [29] with 100000 simulations showed a low likelihood of these results arising by chance, with *P* < 10^−06^ for Graft versus Host disease, *P* ~ 0.002 for Type 1 Diabetes and Asthma and *P* ~ 0.007 for Allograft rejection (Additional file 1: Table S2).

Despite the multiple and convincing evidence in favor of the results, we explain below why these are false positives, which is mainly as a consequence of data handling. In the following sections we will explore strategies to avoid such false-positive findings in GSEA.

## Pre-processing the data

Every GSEA method is highly dependent on the input provided. As an example, while PANTHER [6] accepts as input gene symbols without associated *p*-values, MAGENTA, Ingenuity and EVA take as input SNP IDs with *p*-values, obtained, for instance, from GWAS, and use this *p*-value information to calculate proportions of significant signals according to predefined thresholds.

When providing SNPs and *p*-values to GSEA, it is important to check for the LD structure of the data provided, since GWAS arrays and especially gene-centric arrays have dense coverage in regions of particular interest, and lower coverage in other regions of the genome. Such unbalance can lead to artificial enrichment of regions, in case a densely covered region presents an LD block under the *p*-value threshold determined; the opposite can also happen, and a potentially important region may be lost due to a non-significant LD block in the vicinity. A recent study by Sobota et al. [30] concludes that an r [2] of 0.3 is a reasonable threshold to eliminate redundancy, and our tests corroborate that recommendation, with the original 2,438,671 SNPs narrowed down to 410,986, without losing any locus of the top SNP hits (*P* < 10^−3^).

## SNP-to-gene mapping

Another crucial step is the SNP-to-gene mapping. Recent studies suggest that a 100 kb window gives on average one mapping per SNP [31]. Using the same window of 100 kb, we obtained 2.6 mappings per SNP on average, with 1,801,727 SNPs mapped 4,762,714 times), at the cost of missing ~600 k SNPs, which are probably located in gene deserts.

Larger windows can unveil promoter and enhancer mappings, which are commonly within 500 kb regions of the SNP [32–34] (although enhancers can be found further away [35]). Also, more complex regions, such as MHC, may have SNPs in LD separated by over 3 M bases [36], and rare variant effects may be found up to 2.5 M bases away from the tag SNP [37, 38].. We do not recommend large windows for SNP-to-gene mapping, as the number of mappings becomes intractable: our tests showed an average of 9 mappings per SNP with a 500 kb window, with 2,241,172 SNPs being mapped 20,159,139 times. For a 1 Mb window, 2,387,544 SNPs were mapped 34,793,184 times, giving an average of 14.6 mappings per SNP, most of them likely to be false mappings regarding LD structure (encompassing multiple unrelated genes, due to the mapping based only on distance). Such extensive mapping may lead to overplay of the effects of a single SNP in multiple genes, and if it happens to be a highly significant gene, it could drive the overrepresentation of a whole gene set [39]. These patterns remain even after LD clumping (best proxy method implemented in Plink [40], independent of *p*-value), with 301,509 SNPs mapped 830,835 times (average 2.8 mappings per SNP) for a 100 kb window, and 367,854 SNPs mapped 3,542,909 times (average 9.6 mappings per SNP) using a 500 kb window. A 100 kb window thus seems reasonable to obtain the most manageable ratio of “true” mappings, under an assumption that the nearest genes are most likely the ones affected by a variant (although it is arguably not always the case).

## Gene sets

Efforts to represent collections of gene sets representing biological pathways have been an active research topic for the past 20 years [9, 41, 42]. In general terms, each pathway contains a set of genes that contribute to a certain metabolic process or biological function, obtained from a multitude of experiments. Each of these experiments and databases has its own level of confidence, which may lead to spurious results if not taken into account.

To explain this point, we describe an example. MSigdb [15] has collections of signature databases available that were composed in 2005, including KEGG [10]. The definition for Type I diabetes (T1D) from KEGG at MSigdb contains 44 genes, including 20 *HLA* genes. While it is striking that so many HLA-related genes play a role in development of T1D at the same time, it is not surprising that inflammatory processes are involved in the pathogenesis of this disease. However, KEGG updated its definition of the T1D disease pathway with the most recent research findings, and it currently consists of 22 genes, only three of which are *HLA* genes [43]. This new set is arguably more precise, and is likely to lead to different results when used as definition of a GSEA, although MSIGdb has not been updated since. Adriaens et al. [44] suggest that Reactome should be taken as an initial database for analyses, because of its curation system, which gives more reliable definitons. Other methods, such as Ingenuity [8], also have curation systems, to improve the confidence on the results.

Recently, Frost et al. [45] addressed the problem of generality of pathways, i.e., multiple genes in the same region are assigned to a pathway due to the knowledge one of these genes is involved in the process, but there is no certainty which. These researchers used gene expression data to score how significant each gene of a pathway definition actually is, and were able to narrow down pathways to represent more meaningful biological processes. We believe this is a necessary step to help reduce false positive findings in GSEA.

## Test case: CARDIoGRAM and C4D

In the GWAS field, the high number of false-positive findings of the early studies [46–48] has led to a very stringent *p*-value significance threshold and a mandatory replication step in independent samples [49, 50]. We believe that the GWAS protocol should be used to GSEA as well. In order to test our method, we decided to use a well-established meta-analysis on CAD from the CARDIoGRAMplusC4D consortium [51]. Coronary ARtery DIsease Genome wide Replication and Meta-analysis (CARDIoGRAM) is a consortium of 14 cohorts with multiple recruitment criteria, assessing coronary artery disease (CAD) status of over 80,000 individuals of European ancestry. The Coronary Artery Disease Genetics (C4D) is a similar consortium, established to assess CAD status of over 30,000 patients from four different cohorts of European and south Asian ancestry. Results of independent meta-analyses from CARDIoGRAM [25] and C4D [26] are available online, with GWAS chip and imputed SNPs from CARDIoGRAM (~2.5 M SNPs) but only GWAS chip results from C4D (~500 k SNPs). In order to level both datasets we performed imputation using DISTMIX [52] on C4D, and after imputation we had most of the same SNPs in both datasets. After 1000Genomes-based clumping of an unrelated phenotype, metabolic burden (methods described in Tragante et al. [53]) (r^2^ > =0.3), both datasets had over 400,000 SNPs for pathway analysis. We used a 100 kb window around each SNP to map them to genes in the vicinity. Finally, we used a combination of MSigdb gene sets C2 (curated gene sets) and C5 (Gene Ontology gene sets), which include REACTOME, KEGG and Gene Ontology terms.

Using the full original CARDIoGRAM as the discovery set (given its bigger sample size), we obtained 224 pathways with *P* < 0.05, 69 pathways with *P* < 0.01 and 13 pathways with *P* < =0.001. With C4D for replication, we obtained 21 of the 224 discovery pathways under the *P* < 0.05 threshold, eight of those with *P* < 0.01 and two below *P* < =0.001. With the clumped datasets, we obtained 250 pathways with *P* < 0.05, 68 pathways with *P* < 0.01 and 15 pathways with *P* < =0.001. Replication in C4D reached 20 of 250 discovery pathways with *P* < 0.05, nine of those with *P* < 0.01 and three with *P* < =0.001. Moreover, eight pathways are significant for both CARDIoGRAM and C4D, original sets and clumped sets. The main pathway identified is Biocarta’s Acute Myocardial Infarct pathway, which is an on-target result. Other pathways are related to lipid and platelet metabolisms, which are also directly related to CAD and MI as risk factors (Additional file 1: Table S3). One advantage of the clumped results over the original datasets is the convergence between gene *p*-values of both datasets. While for the original datasets 30 out of 56 *bona fide* genes, (i.e., with genome-wide significant SNPs) have either *P* < 0.05 or *P* > 0.05 on both datasets, for the clumped datasets, 38 out of these 56 genes are convergent (Additional file 1: Table S4).

## Rerunning the heart failure phenotype with complete preprocessing

Using CHARGE GWAS and imputed data (~2.5 M SNPs) as the discovery set (due to the bigger sample size), we identified 54 pathways with *P* < 0.01, 3 of these with *P* < =0.001 (Additional file 1: Table S5), which were the same three pathways that were significantly associated after FDR correction (q = 0.05).

We used two independent cohorts to validate our findings: GoDARTS (~240 k exome chip SNPs) and PREVEND (GWAS chip and imputed ~2.5 M SNPs). We used the following parameters for discovery and replication: clumped SNP lists with the lead signal from an LD block of r^2^ > =0.3, a 100 kb window for mapping SNPs to genes, and MSigdb gene sets C2 (curated gene sets) and C5 (Gene Ontology gene sets). Replication in PREVEND resulted in 33 pathways with *P* < 0.01, 3 of these with *P* < 0.001 (Additional file 1: Table S6) and significant after FDR correction (q = 0.05). However, there was no overlap between any of these pathways.

We then decided to increase our SNP-to-gene mapping window, to 500 kb, keeping the other parameters the same. We then obtained 74 pathways with *P* < 0.01, 19 of those with *P* < 0.001 (Additional file 1: Table S7) and significant after FDR correction (q = 0.05) with the CHARGE dataset, and 57 pathways with *P* < 0.01, 10 of those with *P* < 0.001 (Additional file 1: Table S8) and significant after FDR correction (q = 0.05). Two pathways overlapped between these two sets of results: NIKOLSKY_BREAST_CANCER_16P13_AMPLICON, consisting of 120 genes, and NIKOLSKY_BREAST_CANCER_8Q23_Q24_AMPLICON, consisting of 158 genes. We then investigated how many of these genes have low *p*-values for both datasets. Surprisingly, none of the 120 genes of the first pathway were significant in both datasets, and only three out of the 158 genes of the second pathway had a *P* < 0.05 in the first pathway, which is lower than expected by chance (binomial *P* = 0.03).

The possible advantage of the known SNP-to-gene mapping of the Exome Chip data in their use in GoDARTS is undermined by the fact that there are few SNPs per gene after clumping, leading to imprecise statistics per gene. Therefore, we could not make use of the Exome Chip results.

## Discussion

**Fig. 1:**
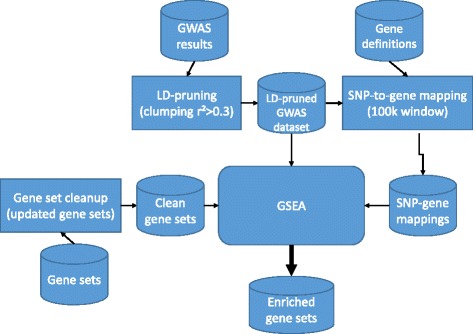
Pipeline of a GSEA. Preprocessing is essential for all the input of the system

Gene set enrichment analysis is a strategy to bring insight into biological mechanisms that lead to disease. Experience from years of GWAS analyses has shown that effect sizes of genetic variants identified are small. Grouping these variants into biologically meaningful pathways, such as is done by GSEA, seems to be a potential alternative to gain power and identify true associations. A very detailed setup is necessary, however, to obtain reliable and reproducible results. From our example, we have shown that false-positive results can be found and even replicated without proper data handling. It is important to have well-defined pipelines for quality control, in order to avoid publishing false-positives, re-working and delay of scientific development. We provide in Fig. 1 a diagram of an ideal GSE analysis, with all pre-processing steps we described in the paper.

The approach described proved successful for CAD/MI. Using CARDIoGRAM and C4D, two big consortia that provide detailed meta-analysis results, we were able to identify significant pathways that replicated on both datasets, and results are directly related to the phenotype. It is also worth noting that with the clumping, regions of high coverage of non-significant SNPs are cleaned up, giving a better estimate of the contribution of each gene in the phenotype. We exemplify this effect with the higher convergence rate between CARDIoGRAM and C4D for genes mapped from the genome-wide significant loci of the phenotype (38 out of 56, binomial *P* = 0.01).

Several factors may have played a role in the absence of positive results for the heart failure phenotype after data QC. The phenotype definition is one of them. Given the complexity of the heart failure syndrome with different phenotypes between distinct ethnic cohorts and different etiologies, genome wide association studies on incident HF can be hampered by heterogeneity. For particularly heterogeneous phenotypes such as HF, large sample sizes are necessary to overcome the noise intrinsic to the data. Ideally, a better phenotyping and subsetting of the individuals might lead to a more clear separation of the genotypes, and results would be clearer with the sample sizes of current studies (~10^5^ – 10^6^ individuals). Of note, a similar GSEA setup in terms of SNP-to-gene mapping and mapping window size, conducted by Ghosh et al. [31], in the domain of coronary artery disease (CAD) (a less heterogeneous phenotype), with the aforementioned sample size (~10^5^ – 10^6^ individuals), succeeded in replicating pathways from Reactome, at a *P* < 0.05 level. Their results led to new hypotheses on mechanisms of CAD that make biological sense demonstrating that a more homogeneous phenotype can lead to successful GSE analyses.

Furthermore, there is a predominance of immunological and cancer-related pathways among the pathways available in MSigdb, coming from multiple papers on the subcategory C2. Pathways with “cancer” as part of their names correspond to 9.4% of all pathways in this subcategory (446 out of 4725), and the terms “lymphoma”, “myeloma” and “blastoma” bring an extra 268 results (5.7%). A more balanced list of reference pathways may help identify biologically relevant processes for traits and diseases in other fields.

The SNP-to-gene mapping requires further improvement. In our tests, a window of 100 kb around the SNP position seems to provide the best ratio of mappings per SNP, in terms of biologically plausible mappings (lower windows would reduce the SNPs mapped up to over half of the input). We note, however, that this is not a final solution; methods that integrate functional assays with LD blocks could help narrow down the number of possible mappings, making the mapping more precise. Furthermore, there is the need to avoid multiple mappings of a single significant SNP to genes, as it could drive the overrepresentation of a whole gene set [39]. This mistake has led to retraction of a manuscript, since the main result had been inflated by the same SNP being mapped to eight genes in the same GO term [54]. This is a hypothesis to be investigated in the future, by making use of the current deluge of data being produced for functional analysis, such as ChIP-seq, RNA-seq, 4C-seq and eQTLs to provide a precise SNP-to-gene mapping and limit noise. Moreover, new computational methods that estimate the uncertainty of the potential causal, nearby gene(s) into the enrichment analysis could be very useful for appropriate significance assessment.

## Conclusions

We believe that GSEA is particularly interesting, for example, in domains with high heritability and low penetrance, such as glucose levels, since multiple mechanisms may be influencing the outcome. It may also be useful for phenotypes in which the known genetic variants explain a low percentage of the phenotypic variance so far, such as blood pressure, because the individual effect of the SNPs associated is small, and grouping small effect SNPs can help in finding novel pathways. GSEA may also help identify cross-ethnic analyses, since different functional SNPs from the same gene without consistent effect across different populations may aggregate at the pathway level, making pathways more likely to replicate than individual SNPs.

GSEA methods have been gaining momentum as part of the GWAS discovery pipeline, and we believe that, with the appropriate setup and configuration, they will help elucidate biological mechanisms underlying phenotypes and diseases.

## Additional files

Additional file 1: Table S2. Supplementary file. Supporting information and analyses results. (XLSX 45 kb)

Additional file 2: Figure S1. Overlapping genes among significant pathways for hearf failure before data processing. (PDF 145 kb)

## Abbreviations

4C-seq: Circularized chromosome conformation capture sequencing
C4D: The Coronary Artery Disease consortium
CAD: Coronary artery disease
CARDIoGRAM: Coronary Artery DIsease Genome wide Replication and Meta-analysis
CHARGE: Cohorts for Heart and Aging Research in Genomic Epidemiology
ChIP-seq: chromatin immunoprecipitation sequencing
eQTL: Expression quantitative trait locis
GO: Gene ontology
GoDARTS: Genetics of Diabetes and Audit Research Tayside Study
GSEA: Gene set enrichment analysis
GWAS: Genome wide association study
HF: Heart failure
HLA: Human leukocyte antigen
KEGG: Kyoto Encyclopedia of Genes and Genomes
LD: Linkage disequilibrium
MAGENTA: Meta-Analysis Gene-set Enrichment of variant Associations
MHC: Major histocompatibility complex
MI: Myocardial infarction
MSigDB: Molecular Signatures Database
PANTHER: Protein Analysis Through Evolutionary Relationships
PREVEND: Prevention of Renal and Vascular Endstage Disease
RNA-seq: RNA sequencing (also known as whole transcriptome shotgun sequencing)
SNP: Single nucleotide polymorphism
