# Supplementary material for: Gene Set Enrichment Analyses: lessons learned from the heart failure phenotype
Source: BioData Min. 2017 May 26;10:18. doi: 10.1186/s13040-017-0137-5 (PMC5446754; doi:10.1186/s13040-017-0137-5)
Supplement: Supplementary file 2 — Overlapping genes among significant pathways for hearf failure before data processing. (PDF 145 kb) [file 13040_2017_137_MOESM2_ESM.pdf]

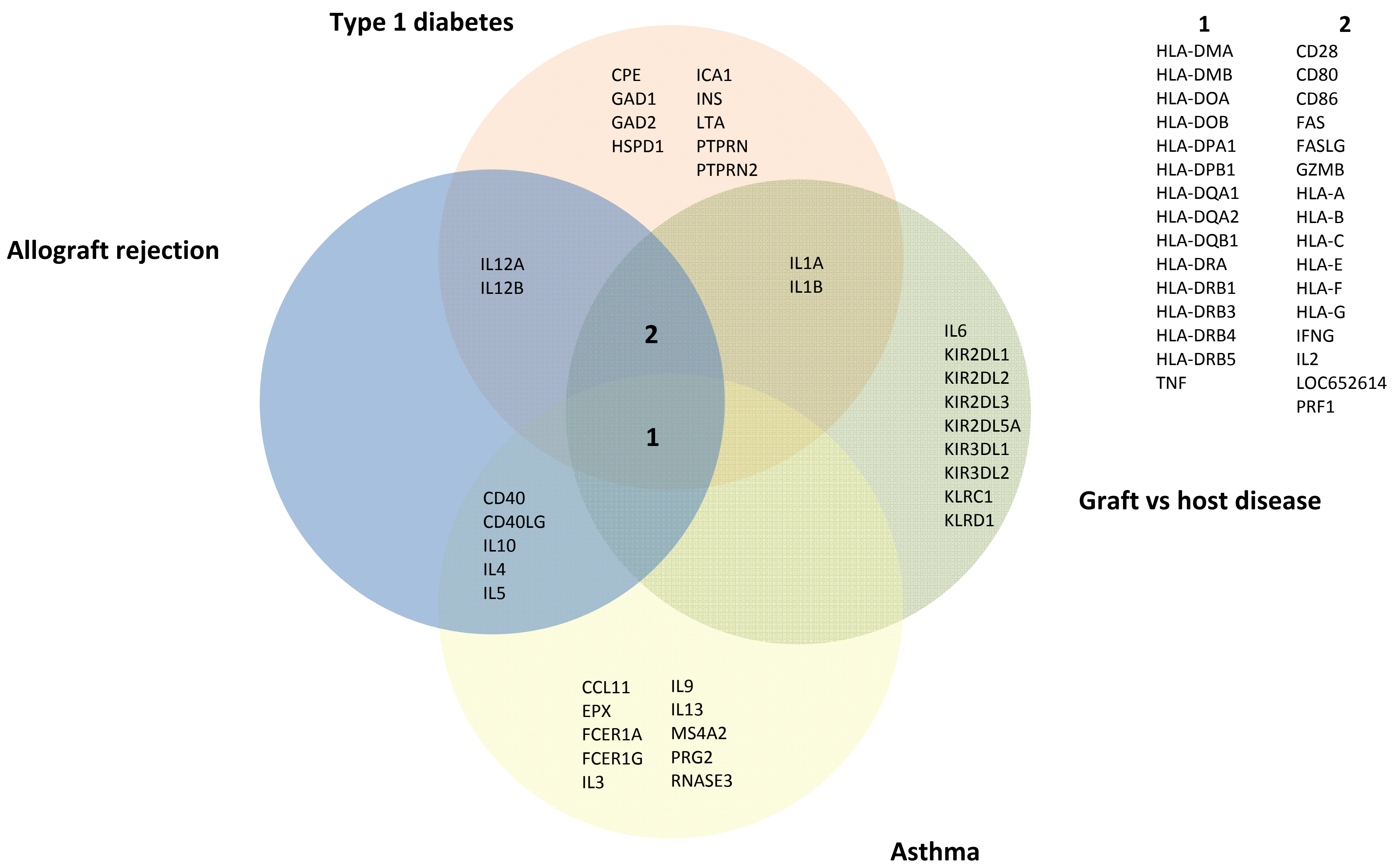


**Supplementary Figure 1.** Overlapping genes among significant pathways for hearf failure before data processing.
